# Idiopathic Uveitis and Familial Mediterranean Fever: Is There Any Relationship?

**DOI:** 10.1155/2014/238931

**Published:** 2014-01-30

**Authors:** Farhad Salehzadeh, Ozra Yasrebi, Mahsa Hosseini Khotbesara, Maryam Hosseini Khotbesara

**Affiliations:** ^1^Pediatric Department, Ardabil University of Medical Sciences (ARUMS), Ardabil 56157, Iran; ^2^Department of Pediatrics, Ardabil University of Medical Sciences (ARUMS), Bouali Hospital, Iran

## Abstract

*Introduction*. Familial Mediterranean fever (FMF) is an auto-inflammatory disease characterized by attacks of fever and polyserositis. FMF is often associated with other autoimmune diseases such as rheumatoid arthritis, polyarteritis nodosa (PAN), and Behcet. Uveitis is an inflammatory process caused by underlying infectious and inflammatory disorders. This study investigates the probable relationship between idiopathic uveitis and FMF. *Methods*. Patients with idiopathic uveitis were analyzed for the 12 most common MEFV mutations (P369S, F479L, M680I(G/C), M680I(G/A), I692del, M694V, M694I, K695R, V726A, A744S, R761H, E148Q) by a reverse hybridization assay (FMF StripAssay,Vienna lab,Vienna, Austria). *Results*. 12 patients with idiopathic uveitis were enrolled in this study. 10 of them were female. The youngest patient was a 7-year-old child and the oldest was 57. The most common complaints of patients were blurred vision and then eye redness. One patient was heterozygous for R761H. Genetic analysis of the 12 most common MEFV mutations in the patients with idiopathic uveitis didnot have any positive results. *Conclusion*. According to the analysis of the 12 most common MEFV gene mutations, FMF is not an underlying cause of idiopathic uveitis. On the other hand, uveitis merely could not be the first presentation of FMF.

## 1. Introduction

FMF is a Mendelian autosomal recessive disease that is highly prevalent in the Mediterranean population [1, 2] characterized by attacks of fever and painful manifestations [3]. The disease is characterized mainly by fever, abdominal pain, arthritis, and erysipelas [4]. The MEFV gene with more than 240 mutations is responsible for FMF [5].

Uveitis is defined as an inflammation of the uveal tract (the iris, ciliary body, choroid) [6, 7] and may involve the optic nerve, sclera, retina, and vitreous humor [8]. Idiopathic uveitis is a condition in which no cause is identified. The uveitis was classified in different ways, based on the anatomical location of the inflammation: anterior, intermediate, posterior, and panuveitis and based on the course of uveitis: acute, chronic, and recurrent, and on etiology it was classified as infectious, autoimmune, and idiopathic, [6, 8]. Uveitis is a very serious and potentially blinding disease [9]. In the uveitis, different patterns of etiology, disease presentation, and progression are associated with difference of race and ethnicity [10].

In Ozdal study, ocular toxoplasmosis was the main cause of infectious uveitis. Other causes of infectious uveitis were herpes, toxocara, and brucella [11]. One common disease that associated with idiopathic uveitis, is Behcet's disease. This disease has a low proportion as an etiologic agent in nonendemic areas but it is one of the common causes of uveitis in endemic areas. Behcet has been observed in 0.5–2.2% of the patients with childhood-onset uveitis in nonendemic areas [12–14]. But in a study in Turkey, Behcet's disease was reported to be the cause of 16.5% of cases with uveitis [15].

FMF is often associated with other autoimmune diseases such as ankylosing spondylitis, rheumatoid arthritis, PAN, Behcet and MS; all are chronic progressive with unknown etiology [16].

Ophthalmological manifestations of FMF are few and include retinal colloid-like bodies, panuveitis, anterior uveitis, scleritis, episcleritis, and papillitis. Michaelson's team published the first report of eye manifestations in FMF in 1959 [17].

According to the influence of geographical factors in the etiology of idiopathic uveitis and since FMF is highly prevalent in this area [2], the present study was designed to answer these questions, does FMF play a role in the etiology of idiopathic uveitis? And whether uveitis would be the only manifestation of FMF?

## 2. Material and Methods

12 patients with idiopathic uveitis were enrolled in this study. All patients had no underlying cause for the uveitis on the basis of clinical and paraclinical evaluation (complete blood count CBC diff, Erythrocyte sedimentation rate (ESR), C reactive protein (CRP), Tuberculin test (PPD), Renal Tests, Liver Functions, ANA (profile), RF, HLA-B27, HLA-B5-B51, Angiotensin converting enzyme (ACE), Chest X-Ray, ANCA, Lyme (Ig), Toxoplasma (antibody), Borrelia (antibody), Wright, 2Mercapto ethanol) when they enrolled in this study.

All the patients were screened for 12 common MEFV mutations by a reverse hybridization assay (FMF StripAssay, Vienna lab, Vienna, Austria) according to the instructions provided by the manufacturer. About 10 mL of peripheral blood was used for extracting DNA by boiling-based method. In a first step, exons 2, 3, 5, and 10 were amplified for each patient in a single, multiplex PCR, with primers supplied by the noted RDB kit. The thermocycling program of amplification was 35 cycles including (94 c for 15 sec, 58 c for 45 sec, and 72 c for 45 sec) and a final extension at 72 c for 7 min. Agarose electrophoresis revealed the accuracy of amplification by detecting four amplified DNA fragments including 206, 236, 295, and 318 bp. Biotinylated PCR products were selectively hybridized to a test strip presenting a parallel array of allele-specific oligonucleotide Probes. Thereby, the twelve common mutations E148Q in exon 2, P369S in exon 3, F479L in exon 5 and M680I (G/C), M680I (G/A), I692del, M694V, M694I, K695R, V726A, A744S, and R761H in exon 10 were determined.

Statistical analysis was performed using SPSS V 15.0. A *P* value of 0.05 was accepted as statistically significant.

## 3. Results

Among 12 patients, 10 of them were female and the remaining 2 were male. The youngest patient was a 7-year-old child and and the oldest was 57. The most common complaints of patients were blurred vision in 7 cases. Among these 7 patients, three had bilateral blurred vision, two had blurred vision in left eye, and two had blurred vision in right eye. The most commonly associated symptoms were gastrointestinal and musculoskeletal symptoms. The MEFV genetic analyses were performed in these 12 patients. No mutations were found in 11 patients (91.66%). One patient was found to be heterozygous for Wt/R761H (Table 1).

## 4. Discussion

FMF is often associated with other autoimmune diseases such as hepatitis, PAN, Behcet, and Multiple Sclerosis (MS). The association of familial Mediterranean fever with juvenile idiopathic arthritis (JIA) or ankylosing spondylitis and Systemic Lupus (SLE) has been described in several previous reports [9]. Michaelson reported eye manifestations of FMF as colloid-like bodes, in 56% of their Jewish patients (compared to 11% of the nonJewish ones). Subsequent studies from the same center of a larger group of patients yielded a lower prevalence (20%), and a later review of 50 Armenian and Arab patients with FMF revealed colloid-like bodies in only 4. Consequently, ophthalmic manifestations were dropped from the clinical criteria of FMF and are only used as findings to rule out this diagnosis [18].

In 1982, Yazici and Pazarli reported a case of anterior uveitis in a woman with FMF who later developed episcleritis. Several years later, four additional cases were described, making a total of five reports of seven cases of uveitis and episcleritis in FMF patients. Overall, four cases of episcleritis, two cases of panuveitis, and two cases of anterior uveitis associated FMF have been reported [18]. On the basis of these studies, looking for the relationship between varieties of eye involvement such as uveitis and FMF will not be unreasonable. Although these studies discussed different eye involvements in FMF patients, we investigated FMF as a background of idiopathic uveitis.

Recently, genetic criteria have been used for the diagnosis of FMF. According to genetic study and analysis of 12 common MEFV gene mutations in the present study, it seems that when we encounter idiopathic uveitis in an especial area with high prevalence of FMF, it could not be considered as an underlying cause of idiopathic uveitis, although we need all mutations (nearly 250 mutations) of MEFV genes analysis to confirm this result. On the other hand, these results showed that uveitis merely could not be probably the only and first presentation of FMF.

## Supplementary Material

Supplementary file describes about the profile of patients, the main symptom, age, sex and MEFV gene among patients.

## Figures and Tables

**Table 1:** Patients' findings.

No.	Sex	Age (yr)	Main symptom	Other symptoms	Age of onset (yr)	Duration of symptoms (yr)	MEFV gene
1	F	25	Redness	Blurred vision	22	3	Wt/wt
2	F	39	Redness	Blurred vision	34	5	Wt/wt
3	F	28	Blurred vision	Headache	24	4	Wt/wt
4	F	20	Blurred vision	—	17	3	Wt/wt
5	M	12	Blurred vision	—	6	6	Wt/wt
6	F	57	Blurred vision	Pain	54	3	R761H/wt
7	F	23	Blurred vision	—	21	1.5	Wt/wt
8	F	19	Blurred vision	—	15	4	Wt/wt
9	F	22	Blurred vision	—	19	3	Wt/wt
10	F	7	Accidental	—	5	2	Wt/wt
11	F	31	Redness	Pain	21	10	Wt/wt
12	M	14	Redness	Blurred vision	2	1.5	Wt/wt
