# Supplementary material for: Idiopathic Uveitis and Familial Mediterranean Fever: Is There Any Relationship?
Source: Autoimmune Dis. 2014 Jan 30;2014:238931. doi: 10.1155/2014/238931 (PMC3926436; doi:10.1155/2014/238931)
Supplement: Supplementary file 1 — Supplementary file describes about the profile of patients, the main symptom, age, sex and MEFV gene among patients. [file 238931.f1.docx]

| line | Sex | Age  yr | Main symptom | Other symptoms | Age of onset(yr) | Duration of symptoms (yr) | Genetic analysis |
| --- | --- | --- | --- | --- | --- | --- | --- |
| 1 | F | 25 | Redness | blurred vision | 22 | 3 | Wt/wt |
| 2 | F | 39 | Redness | blurred vision | 34 | 5 | Wt/wt |
| 3 | F | 28 | blurred vision | Headache | 24 | 4 | Wt/wt |
| 4 | F | 20 | blurred vision | ---- | 17 | 3 | Wt/wt |
| 5 | M | 12 | blurred vision | ---- | 6 | 6 | Wt/wt |
| 6 | F | 57 | blurred vision | Pain | 54 | 3 | R761H/wt |
| 7 | F | 23 | blurred vision | ---- | 21 | 1.5 | Wt/wt |
| 8 | F | 19 | blurred vision | ---- | 15 | 4 | Wt/wt |
| 9 | F | 22 | blurred vision | ---- | 19 | 3 | Wt/wt |
| 10 | F | 7 | Accidental | ---- | 5 | 2 | Wt/wt |
| 11 | F | 31 | Redness | Pain | 21 | 10 | Wt/wt |
| 12 | M | 14 | Redness | blurred vision | 2 | 1.5 | Wt/wt |
